# Benchmarking large language models for clinical data extraction from Portuguese medical notes in a university hospital

**DOI:** 10.1590/0102-311XEN145025

**Published:** 2026-07-31

**Authors:** Breno Gabriel Araújo Sampaio de Jesus, Tomaz Castrillon Figueiredo, Clariele de Almeida Pereira, Bruno Henrique Lira Anjos, Ney Cristian Amaral Boa-Sorte, André Rodrigues Durães, Carlos Brites, Eduardo Martins, Leandro Maciel Almeida, Cleber Zanchettin, Vinícius de Oliveira Menezes, Marcos Antônio Dórea Machado

**Affiliations:** 1 Hospital Universitário Professor Edgard Santos, Universidade Federal da Bahia, Salvador, Brasil.; 2 Nuclearis, Recife, Brasil.; 3 Centro de Informática, Universidade Federal de Pernambuco, Recife, Brasil.; 4 Hospital das Clínicas, Universidade Federal de Pernambuco, Recife, Brasil.

**Keywords:** Large Language Models, Electronic Health Records, Benchmarking, Modelos de Linguagem de Grande Escala, Registros Eletrônicos de Saúde, Benchmarking, Grandes Modelos de Lenguaje, Registros Electrónicos de Salud, Benchmarking

## Abstract

Extracting structured data from electronic health records (EHRs) remains a major challenge, particularly in non-English and resource-constrained healthcare systems. This study benchmarks multiple large language models (LLMs) for the automated extraction of structured clinical variables from Portuguese-language medical notes under limited computational resources. We evaluated five LLMs (GPT-4o mini, DeepSeek-V3, Mixtral-8x7B, LLaMA 8B, and Qwen-32B) against a manually curated dataset of cardiology and infectiology outpatient records. Models were deployed in quantized versions to optimize computational efficiency. Outputs were compared with human annotations using F1 score, balanced accuracy, and recall. Among the tested models, Qwen-32B achieved the highest performance in both the infectiology domain (balanced accuracy = 0.91 [0.07]) and cardiology domain (balanced accuracy = 0.89 [0.07]). Performance varied by clinical variable, with better results for frequently and consistently documented conditions (e.g., diabetes) and lower accuracy for complex or infrequent variables (e.g., tumors). Extraction time ranged from 0.9 to 24.2 minutes per patient, depending on clinical domain and model. These findings support the feasibility of applying LLM-based natural language processing tools in resource-limited, non-English healthcare settings. Future research should assess emerging high-parameter models and explore additional clinical domains.

## Abstract (pt)

O acesso a dados estruturados de prontuários eletrônicos de saúde (PES) continua sendo um grande desafio, particularmente em sistemas de saúde com recursos limitados e em idiomas diferentes do inglês. Este estudo realiza *benchmarking* de múltiplos grandes modelos de linguagem (LLMs) para a extração automatizada de variáveis clínicas estruturadas a partir de anotações médicas em português brasileiro em um ambiente com recursos computacionais limitados. Avaliamos cinco LLMs (GPT-4o mini, DeepSeek-V3, Mixtral-8x7B, LLaMA 8B e Qwen-32B) em relação a um conjunto de dados anotado e validado manualmente, composto por prontuários de pacientes ambulatoriais de cardiologia e infectologia. Os modelos foram implementados em versões quantizadas para otimizar a eficiência computacional. As respostas (*outputs*) dos modelos foram comparadas com anotações humanas utilizando F1-*score*, acurácia balanceada e sensibilidade (*recall*). Entre os modelos testados, o Qwen-32B alcançou o melhor desempenho tanto no domínio clínico da infectologia (acurácia balanceada = 0,91 [0,07]) quanto no domínio clínico da cardiologia (acurácia balanceada = 0,89 [0,07]). O desempenho dos modelos variou de acordo com a variável clínica, com melhores resultados para condições frequentemente e consistentemente documentadas (p.ex.: diabetes) e menor acurácia para variáveis complexas ou infrequentes (p.ex.: tumores). O tempo de extração variou de 0,9 a 24,2 minutos por paciente, dependendo do domínio clínico e do modelo de LLM. Esses achados indicam a viabilidade da implementação de ferramentas de processamento de linguagem natural baseadas em LLMs em cenários de saúde com recursos limitados e em idiomas diferentes do inglês. Pesquisas futuras devem avaliar modelos emergentes de grande porte e explorar domínios clínicos adicionais.

## Abstract (es)

El acceso a los datos estructurados de las historias clínicas electrónicas (HCE) continúa siendo un gran desafío, especialmente en los sistemas sanitarios con recursos limitados y en idiomas distintos del inglés. Este estudio realiza *benchmarking* de varios modelos de lenguaje (LLMs) para la extracción automatizada de variables clínicas estructuradas a partir de notas médicas en portugués brasileño en un entorno con recursos informáticos limitados. Se evaluaron cinco LLMs (GPT-4o mini, DeepSeek-V3, Mixtral-8x7B, LLaMA 8B y Qwen-32B) con base en un conjunto de datos anotado y validado manualmente, compuesto de historias clínicas de pacientes ambulatorios de cardiología e infectología. Los modelos se implementaron en versiones cuantificadas para optimizar la eficiencia computacional. Las respuestas (*outputs*) de los modelos se compararon con las notas humanas mediante el uso de F1-*score*, de la precisión equilibrada y de la sensibilidad (recall). Entre los modelos probados, Qwen-32B logró el mejor rendimiento tanto en el dominio clínico de la infectología (precisión equilibrada = 0,91 [0,07]) como en el dominio clínico de la cardiología (precisión equilibrada = 0,89 [0,07]). El rendimiento del modelo tuvo variaciones según la variable clínica, con mejores resultados para afecciones documentadas con frecuencia y consistencia (por ejemplo, diabetes) y menor precisión para variables complejas o infrecuentes (por ejemplo, tumores). El tiempo de extracción varió de 0,9 a 24,2 minutos por paciente dependiendo del dominio clínico y del modelo de LLM. Estos hallazgos indican la viabilidad de implementar herramientas de procesamiento de lenguaje natural basadas en LLMs en entornos de atención médica con recursos limitados y en idiomas distintos del inglés. Las investigaciones futuras pueden evaluar modelos emergentes a gran escala y explorar dominios clínicos adicionales.

## Introduction

Extracting structured and standardized data from electronic health records (EHRs) remains a major challenge, as most clinically relevant information is stored in unstructured text formats. Manual extraction is time-consuming and prone to errors. In this context, natural language processing (NLP) techniques enable automated and scalable data extraction from narrative clinical notes ^1^ ^,^ ^2^ ^,^ ^3^ ^,^ ^4^ ^,^ ^5^ ^,^ ^6^. By leveraging NLP, we can optimize clinical and research workflows, enhancing clinical decision-making ^1^ ^,^ ^2^ ^,^ ^7^ ^,^ ^8^.

NLP has demonstrated remarkable performance in data extraction across various medical domains. For instance, Choi et al. ^9^ reported an 87.7% overall accuracy in extracting clinical data from 2,931 ultrasound and pathology reports related to breast cancer, while another study ^10^ achieved 90% accuracy in extracting data from 91,505 reports. Nath et al. ^8^ achieved 94.1% precision in extracting echocardiogram data, and Song et al. ^11^ reported 99.6% accuracy in identifying 10 gastric diseases from esophagogastroduodenoscopy reports.

While traditional NLP approaches, such as rule-based systems or supervised machine learning models (e.g., Conditional Random Fields - CRF - and Recurrent Neural Networks with CRF layers), have demonstrated proficiency in information extraction tasks within specific domains ^8^ ^,^ ^9^ ^,^ ^10^ ^,^ ^11^, the emergence of large language models (LLMs) introduces a distinct paradigm. Pre-trained on vast textual corpora, these models offer the potential for zero-shot or few-shot generalization ^12^, mitigating the need for large volumes of task-specific annotated data, which is a recurrent challenge in resource-constrained healthcare settings such as the Brazilian public health system. Furthermore, LLMs’ capacity to capture complex contextual and semantic nuances ^8^ ^,^ ^9^ ^,^ ^10^ may help overcome limitations of previous models in handling the variability and lack of structure inherent to clinical narratives. Despite recent advances, few studies have evaluated the feasibility of LLMs in low-resource, non-English healthcare systems. Public hospitals in Brazil, for instance, often operate under heterogeneous workflows, unstructured clinical documentation, and limited computational infrastructure.

Moreover, the use of clinical notes in Portuguese presents an additional linguistic barrier, as most pre-trained LLMs are optimized for English. Therefore, this study conducts a comprehensive benchmarking of multiple LLM-based NLP methods for extracting structured data from free-text Portuguese clinical notes in a large university hospital. This study is distinguished by its systematically assessment of quantized LLMs, a critical aspect for operational viability in environments with limited computational infrastructure, and by its focus on Brazilian Portuguese, an underrepresented language in healthcare-related LLM research.

## Methods

### Patients

De-identified data were retrieved from the EHRs of the cardiology and infectiology outpatient clinics at Professor Edgard Santos Universitary Hospital, Federal University of Bahia (HUPES/UFBA, acronym in Portuguese), Salvador, Bahia State, Brazil. The dataset includes clinical records from 2019 to 2024. The study was approved by the HUPES/UFBA Ethics Research Committee (CAAE: 58972522.7.0000.0049), which waived the requirement for informed consent.

All clinical notes used in this study were fully de-identified prior to analysis. The hospital information system structures patient records with metadata fields (e.g., name, date, note type) and a separate free-text field for clinical findings. Only the de-identified free-text content, without any associated metadata or identifiers, was provided to the research team in .csv format. Patient personally identifiable information (PII) and protected health information (PHI) were removed through the institutional data privacy workflow, and each record was assigned a unique study ID generated by the data warehouse.

The open-source LLMs were executed locally on a research server administered by the study team, ensuring controlled deployment. For the API-based using proprietary model, data submission complied with all privacy safeguards. According to the platform’s usage policy, no information is stored or used for training when privacy controls are enabled. Access to the proprietary API was provided by Nuclearis, a company offering a platform with regulatory clearance from the Brazilian Health Regulatory Agency (Anvisa, acronym in Portuguese) for AI-assisted software. Nuclearis enables secure access to third-party APIs in its integrated platform. In this study, Nuclearis collaborated with the hospital’s Health Technology Assessment Department and Health Innovation and Technology Management Department to implement and validate a data mining service designed to support researchers in applying advanced AI tools to clinical and translational studies.

### Feature selection

Clinicians with experience in outpatient cardiology and infectiology care identified 14 variables of interest for each domain (Box 1). These variables were selected based on their clinical relevance.

**Box 1:** Features selected for evaluation.

CLINICAL DOMAIN	VARIABLES
Cardiology	Smoking (*tabagismo*); hypertension (*hipertensão*); diabetes mellitus (*diabetes mellitus*); dyslipidemia (*dislipidemia*); family history of diabetes, systemic arterial hypertension, dyslipidemia, or stroke (*história familiar de diabetes, hipertensão, dislipidemia ou acidente vascular encefálico*); family history of coronary artery disease (*história familiar de doença coronariana*); angina (*angina*); myocardial infarction (*infarto agudo do miocárdio*); obesity (*obesidade*); sedentary lifestyle (*sedentarismo*); stroke (*acidente vascular encefálico*); aspirin use (*uso de aspirina*); atrial fibrillation (*fibrilação atrial*); heart failure (*insuficiência cardíaca*)
Infectiology	Fever (*febre*); unintentional weight loss (*perda de peso não-intencional*); cough (*tosse*); diarrhea (*diarreia*); jaundice (*icterícia*); pain (*dor*); seizures (*convulsão*); skin lesions (*lesão de pele*); tumors (*tumores*); dyspnea (*dispneia*); anosmia or ageusia (*anosmia ou ageusia*); illicit drug use (*uso de drogas ilícitas*); diabetes mellitus (*diabetes mellitus*); hypertension (*hipertensão*)

### Clinical note selection and preprocessing

A random sample of 150 subjects per department (cardiology and infectiology), without prior group allocation, was selected for data extraction. To ensure a minimum of 20 positive and 20 negative cases for each variable under analysis, we systematically evaluated the class distribution of the initial random sampling. When the predefined threshold was not achieved, additional targeted searches (i.e., using keywords related to underrepresented variables) were conducted within the institutional database to identify and include specific cases necessary to meet the minimum class balance. Prior to NLP extraction, consecutive notes from the same patients were merged into a single medical record to facilitate cross-sectional analysis.

### Benchmark database

Trained reviewers manually classified the presence or absence of each clinical condition, thereby establishing the benchmark database. Variables were classified as either present (1) or absent (0) based on commonly used Portuguese terms in the clinical notes and domain-specific knowledge of relevant terminology. For example, to identify arterial hypertension, a set of 12 Portuguese expressions was compiled, including “hipertensão arterial sistêmica” (systemic arterial hypertension), “HAS” (acronym of the former expression), “paciente hipertenso” (hypertensive patient), and “pressão arterial elevada” (elevated blood pressure). The key terms were normalized (converted to lowercase and stripped of diacritics) and aggregated into a canonical phrase structure to guide the manual classification process. The same approach was applied to all clinical variables (Supplementary Material; https://cadernos.ensp.fiocruz.br/static//arquivo/supl-e00145025_7960.pdf). This binarization facilitated quantitative comparison with model outputs.

Since notes from different consultations for the same patient were compiled, in cases of conflicting information, the presence of a variable was prioritized. When no information related to a specific variable was identified, it was classified as absent. This strategy for handling conflicting data (i.e., prioritizing presence) and inferring absence from non-mention aligns with common pragmatic approaches in EHR information extraction. However, we acknowledge the inherent limitation that “absence of evidence is not evidence of absence”, as the lack of documentation may stem from factors other than the true absence of the condition. This is a common challenge when relying on secondary EHR data for clinical inference.

### Natural language processing

All model inferences and evaluations were conducted on a local workstation equipped with an NVIDIA RTX 6000 Ada Generation GPU (48 GB VRAM) and 192 GB of system RAM, running Ubuntu 22.04 LTS with Python version 3.12.7 (http://www.python.org). The following LLM models were assessed: DeepSeek-V3 ^13^, Mixtral-8x7B (https://mistral.ai/news/mixtral-of-experts/), LLaMA 8B (https://ai.meta.com/llama/), and Qwen-32B (https://huggingface.co/Qwen). GPT-4o mini ^14^ (Open AI API) was the only model accessed via API and, therefore, was not quantized and free from the computational constraints associated with loading and running the model. These models were selected to represent a diversity of architectures (e.g., mixture-of-experts such as Mixtral and dense models), parameter sizes (8B to 32B for open-source models), origins (proprietary and open-source), and their reported availability for efficient quantization techniques. This selection enabled a comprehensive assessment of the current LLM landscape applicable to computationally constrained scenarios, which is a key consideration for real-world use in many healthcare settings. Computational efficiency was a critical factor in model selection and deployment. To ensure the feasibility for large-scale extraction within constrained computational environments, open-source models were employed in quantized versions. Quantization reduces the numerical representation of model weights, thereby decreasing memory footprint and inference latency and enabling efficient processing of extensive clinical note datasets. Prompt engineering was another critical component (detailed parameters are available in the Supplementary Material; https://cadernos.ensp.fiocruz.br/static//arquivo/supl-e00145025_7960.pdf). To ensure consistent and directly comparable extraction, all models received the same structured prompt template, consisting of: (1) a concise instruction directing the model to complete a predefined JSON schema, which was embedded immediately thereafter; (2) the raw clinical note; and (3) a bullet-point list of representative synonyms and real-world examples for each target variable. Generation settings were identical across models, using greedy decoding (*do_sample=False*).

**Figure 1:**
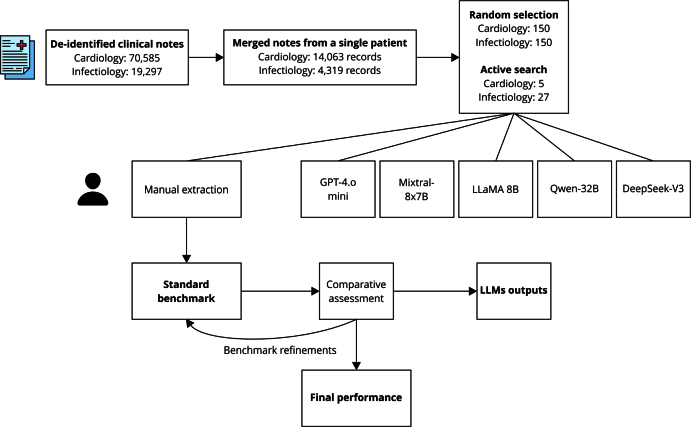
Methodology workflow.

LLMs were applied to the clinical notes to generate a structured database. We conducted consecutive experiments to optimize the prompts. The output of each model was compared with the benchmark. In cases of disagreement between models and the reference, we performed a detailed error analysis, followed by iterative refinements to both the correction of benchmark and prompt engineering (Figure 1).

LLMs: large language models.

The best-performing model for each variable, based on benchmark evaluation, was then applied to the complete database for extraction. Processing time was reported as the mean inference time per patient, aggregating all clinical notes associated with each case. For open-source models, execution was performed locally, and the reported times refer exclusively to the inference stage. For the proprietary API-based model, the values correspond to the total API response time, including communication latency. For all models, preprocessing and anonymization steps were excluded from the reported time measurements.

### Statistical analysis

To evaluate model performance, we compared their results with the reference benchmark to assess its F1 score, balanced accuracy, and recall for each category ^15^ ^,^ ^16^. Recall, also known as sensitivity, was defined as the rate of true positives among all actual positive cases. Balanced accuracy was calculated as the average of recall and specificity, reflecting the model’s ability to correctly classify both positive and negative cases, balancing the weight of different classes. The F1 score represents the harmonic mean of precision and recall, providing a single metric that summarizes overall model performance. All metrics range from 0 to 1, with 1 indicating optimal performance ^15^ ^,^ ^16^. Model performance comparisons were conducted using bootstrapped confidence intervals for mean balanced accuracy (10,000 resamples) and McNemar’s test to evaluate discordant predictions at the patient level. For the McNemar analysis, we constructed 2x2 contingency tables for each clinical variable, comparing the predictions of two models against the gold standard. A significance threshold of p-value < 0.05 was adopted. Descriptive statistics were calculated to summarize the results. Statistical analyses were performed in Python 3.12.7.

## Results

A total of 155 cardiology patients and 174 infectiology patients were analyzed, comprising 1,227 and 1,172 clinical notes, respectively. Benchmark results for balanced accuracy are presented in Table 1 and Table 2 for cardiology and infectiology, respectively. Results for F1 score and recall are presented in the Supplementary Material (https://cadernos.ensp.fiocruz.br/static//arquivo/supl-e00145025_7960.pdf).

**Table 1:** Balanced accuracy for the cardiology dataset.

Parameter	GPT-4o mini	DeepSeek-V3	Mixtral-8x7B	LLaMA 8B	Qwen-32B	Mean
Tobacco use	0.70	0.73	0.67	0.92 *	0.78 **	0.76
Hypertension	0.81 *	0.59	0.56	0.58	0.75 **	0.66
Diabetes	0.90 **	0.66	0.90 **	0.86	0.94 *	0.85
Dyslipidemia	0.70	0.65	0.70	0.72 **	0.81 *	0.71
Family history	0.96 *	0.66	0.80	0.56	0.94 **	0.78
Family history of coronary artery disease	0.91 **	0.83	0.66	0.69	0.95 *	0.81
Angina	0.89	0.78	0.90 **	0.80	0.91 *	0.86
Myocardial infarction	0.92 **	0.84	0.72	0.72	0.93 *	0.82
Obesity	0.90	0.92	0.95 **	0.88	0.96 *	0.92
Sedentarism	0.82	0.90 **	0.84	0.90 **	0.97 *	0.89
Stroke	0.95 *	0.84	0.76	0.78	0.89 **	0.84
Aspirin use	0.96 *	0.78	0.59	0.67	0.94 **	0.79
Atrial fibrillation	0.87	0.91 *	0.84	0.76	0.91 *	0.86
Heart failure	0.69	0.85	0.92 *	0.82	0.84 **	0.83
Mean (SD)	0.86 (0.09) **	0.78 (0.10)	0.77 (0.12)	0.76 (0.11)	0.89 (0.07) *	-

SD: standard deviation.

* The model with the best performance;

** The model with the second best performance.

**Table 2:** Balanced accuracy for the infectiology dataset.

Parameter	GPT-4o mini	DeepSeek-V3	Mixtral-8x7B	LLaMA 8B	Qwen-32B	Mean
Fever	0.93 *	0.56	0.79	0.86	0.90 **	0.81
Weight loss	0.93 **	0.77	0.86	0.79	0.95 *	0.86
Cough	0.94 *	0.81	0.90	0.92 **	0.91	0.90
Diarrhea	0.97 *	0.84	0.88	0.91	0.94 **	0.91
Jaundice	0.96 **	0.81	0.93	0.95	0.99 *	0.93
Pain	0.85 **	0.62	0.79	0.53	0.88 *	0.74
Seizures	0.97 **	0.92	0.95	0.97 **	1.00 *	0.96
Skin spots	0.82 **	0.82 **	0.81	0.80	0.85 *	0.82
Tumors	0.91 *	0.70	0.77	0.87 **	0.87 **	0.82
Dyspnea	0.99 **	0.88	0.99 **	0.96	1.00 *	0.97
Anosmia or ageusia	0.99 *	0.88	0.96	0.98 **	0.96	0.95
Illicit drug use	0.87 **	0.82	0.82	0.75	0.91 *	0.83
Diabetes	0.84	0.73	0.87 *	0.86 **	0.85	0.83
Hypertension	0.62	0.78 *	0.68	0.68	0.76 **	0.71
Mean (SD)	0.90 (0.09) **	0.78 (0.10)	0.86 (0.08)	0.84 (0.12)	0.91 (0.07) *	-

SD: standard deviation.

* The model with the best performance;

** The model with the second best performance.

Among the evaluated models, Qwen-32B demonstrated the highest overall performance in the infectiology domain, achieving a balanced accuracy of 0.91 (0.07) and an F1 score of 0.85 (0.11). However, GPT-4o mini outperformed it in recall, reaching 0.95 (0.05). In the cardiology domain, Qwen-32B again achieved the highest balanced accuracy (0.89 [0.07]) and F1 score (0.83 [0.08]), whereas LLaMA-8B yielded the highest recall at 0.96 (0.04). Model performance varied substantially across variables. For instance, seizure detection reached an F1 score of 1.00 (Qwen-32B), while pain identification remained low across all models, suggesting limitations in interpreting inconsistently documented conditions. In cardiology, hypertension and diabetes were consistently well-detected (mean F1 scores > 0.80), while variables with more context-dependent phrasing, such as family history of coronary disease and aspirin use, showed lower agreement.

Pairwise comparisons of model predictions using McNemar’s test are shown in Supplementary Material (https://cadernos.ensp.fiocruz.br/static//arquivo/supl-e00145025_7960.pdf). Statistically significant discordances were observed between some LLMs. For example, in the infectiology dataset, GPT-4o mini showed significantly fewer discordant errors compared with open-source models (p < 0.05), suggesting greater consistency at the patient level. In the cardiology dataset, Qwen-32B achieved a balanced accuracy similar to that of GPT-4o mini; however, McNemar’s test yielded p < 0.05, indicating that the two models systematically disagreed on which patients were misclassified. This finding highlights that statistical significance may arise from different error distributions, even when overall accuracy levels are equivalent.

Bootstrap resampling (10,000 iterations) showed variability in performance across clinical variables (Supplementary Material; https://cadernos.ensp.fiocruz.br/static//arquivo/supl-e00145025_7960.pdf). Models such as GPT-4o mini and Qwen-32B achieved higher balanced accuracy estimates with narrower percentile intervals, indicating greater stability. Variables with broader intervals showed greater uncertainty, suggesting that performance for these variables was more sensitive to sampling variation. In some cases, non-overlapping percentile intervals indicated consistent performance differences between models, complementing the pairwise findings from McNemar’s tests.

**Figure 2:**
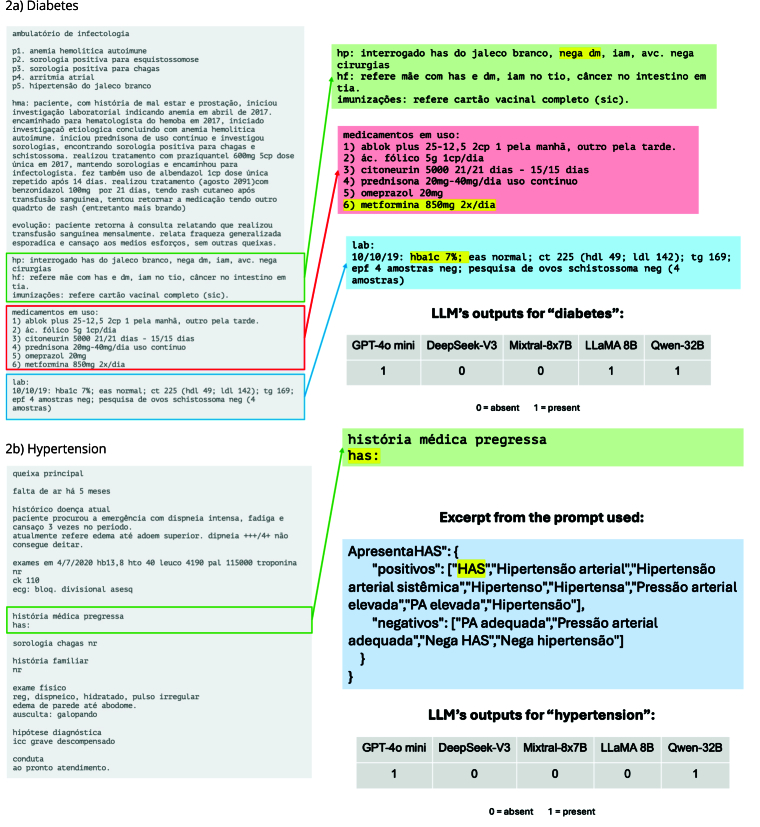
Example of heterogeneous large language model (LLM) performance in the extraction of diabetes and hypertension status from clinical notes.

**Figure 3:**
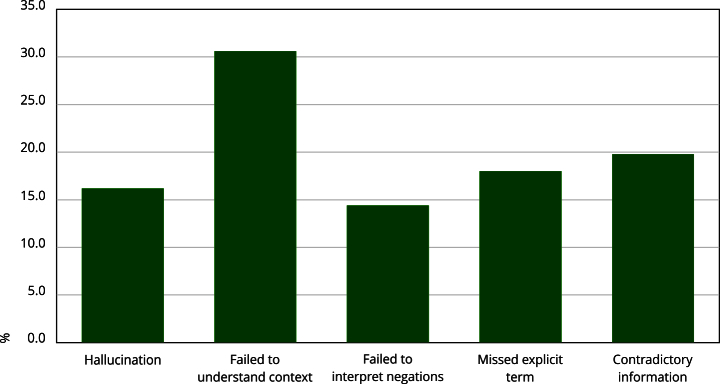
Distribution of error types identified in the subset of discordant cases analyzed.

Figure 2 shows two examples of lexical variation in the clinical notes, illustrating how regional and institutional terminology may affect model generalization. In Figure 2a, the medical history explicitly states “nega DM” (denies diabetes), a regional shorthand referring to diabetes mellitus. Despite this negative, the same note documents chronic metformin use and HbA1c = 7%, findings consistent with diabetes. DeepSeek-V3 and Mixtral-8x7B classified the patient as non-diabetic, whereas GPT-4o mini, LLaMA 8B, and Qwen-32B integrated contextual evidence and correctly identified diabetes. In Figure 2b, the note includes “HAS - hipertensão arterial sistêmica” in the medical history. Despite the explicit mention and prior inclusion of the term in the prompt, only GPT-4o mini and Qwen-32B correctly detected hypertension, while DeepSeek-V3, Mixtral-8x7B, and LLaMA 8B failed to recognize it. To further characterize the model misclassifications, we performed a quantitative error analysis in a representative subset of discordant cases (n = 55), classifying errors into five predefined categories (Figure 3).

The mean processing time per patient (aggregating all associated clinical notes) in the cardiology database was 0.9, 4.3, 0.8, 4.0, and 24.2 minutes for GPT-4o mini, DeepSeek-V3, Mixtral-8x7B, LLaMA 8B, and Qwen-32B models, respectively. For the infectiology database, the mean processing time was 3.0, 3.6, 2.4, 0.7, and 18.7 minutes, respectively. Processing the complete databases required approximately 28 days for cardiology and two days for infectiology when executed sequentially under the described conditions without parallelization.

## Discussion

This study evaluated the performance of LLMs for structured data extraction from unstructured Portuguese clinical notes in a Brazilian university hospital. Our findings support the applicability of these methods in resource-constrained environments and highlight specific challenges and opportunities for their deployment in real-world healthcare systems. To our knowledge, this is the first systematic benchmarking study of multiple LLMs for clinical information extraction from Portuguese-language notes. Our study provides empirical evidence that LLMs can achieve clinically acceptable accuracy in real-world healthcare scenarios beyond the English-speaking context.

### Model performance and variability across clinical variables

LLMs exhibited heterogeneous performance depending on the clinical variable assessed. Models performed well on frequently documented and linguistically straightforward conditions such as diabetes, obesity, and dyspnea. In contrast, variables requiring more nuanced interpretation - such as pain and family history - demonstrated lower performance. This suggests that performance is strongly influenced by how consistently and explicitly information is expressed in clinical narratives, underlining the importance of contextual understanding in clinical LLM applications. Such challenges include accurately interpreting negation scope, resolving complex anaphoric coreferences across sentences or note sections, and distinguishing between confirmed conditions, patient-reported conditions, denied conditions, and differential diagnoses. The inherent variability in documentary practices among healthcare professionals further increases the complexity of the extraction task.

We acknowledge that a larger corpus would enhance statistical robustness. However, expanding the evaluation framework remains part of our ongoing efforts, especially considering that manual labeling of clinical notes is time-consuming and often infeasible at scale. In this study, the main sampling strategy was random. Only a small proportion of cases was selected via keyword searches to ensure minimal representation of minority classes. Specifically, five additional patients (out of 155) were included in the cardiology cohort and 27 additional patients (out of 177) in the infectiology cohort. This targeted inclusion enabled more reliable evaluation of low-prevalence variables, including seizure, anosmia or ageusia, jaundice, and illicit drug use. Additional balancing was also performed for stroke, atrial fibrillation, and family history of coronary artery disease.

### Model-level differences and the impact of quantization

Qwen-32B, an open-source model, achieved the best performance in both domains and was competitive with GPT-4o mini. The use of quantized LLMs, while essential for feasibility in hardware-constrained environments, introduces a trade-off between efficiency and performance. It is known that reducing the numerical precision of model weights (e.g., to 4-bit NF4 for Mixtral-8x7B and DeepSeek-V3, or 8-bit integer for Qwen-32B) can impact the fine-grained representation of linguistic nuances, particularly in tasks requiring high semantic fidelity ^17^. Certain architectures or tasks may be more sensitive to such effects, and the observed performance variability across clinical variables may, in part, reflect quantization-related artifacts. These findings indicate that model size alone is not the key determinant of performance; prompt engineering, alignment with Portuguese-language data, and domain-specific adaptation are also critical.

### Variable-level performance patterns

Performance varied across clinical variables. The iterative process of prompt refinement further emphasized the importance of human oversight in developing and deploying clinical LLM methods. A qualitative error analysis conducted during this iterative process revealed recurrent patterns. False negatives frequently arose from indirect or implicit documentation of conditions in which the model failed to perform the necessary inference. For instance, references to “regular metformin use” without an explicit statement of “diabetes” was occasionally not recognized. False positives were commonly associated with complex negation structures (e.g., “the patient denies a family history of heart disease but reports that the grandfather had a myocardial infarction”), as well as with terms appearing in hypothetical contexts or as part of a differential diagnosis. Terminological ambiguity, despite the inclusion of a synonym list in the prompt, also contributed to errors, particularly for variables with more heterogeneous linguistic representations, such as “tumors” or “family history”. These findings highlight the need for more sophisticated prompt engineering strategies and, potentially, domain-specific fine-tuning to enhance robustness in linguistically challenging cases.

Another factor that may explain significant discordances detected by McNemar’s test, even when average accuracy differences were minimal, is the inherent stochasticity of LLMs. Although prompts and decoding parameters were kept constant, LLMs may still occasionally generate inconsistent outputs or so-called “hallucinations”. While relatively infrequent, such phenomena may contribute to error patterns that differ across models, thereby increasing discordance counts without necessarily altering overall accuracy. This observation underscores the importance of considering both systematic performance differences and the intrinsic variability of generative models in clinical NLP benchmarking.

The bootstrap analysis provided additional insight into the stability of performance estimates. Variables with narrow percentile intervals showed more consistent results across resampling, whereas those with wider intervals demonstrated greater uncertainty, indicating that performance was more sensitive to the specific subset of records sampled. This pattern likely reflects differences in how consistently each variable is documented in the clinical notes.

The bootstrap results also complemented the McNemar’s tests by distinguishing differences in average performance from differences in patient-level error patterns. Some variables showed similar mean accuracy but significant discordance between models, suggesting that errors occurred in different subsets of patients. When percentile intervals did not overlap, the results indicated more robust differences in performance. Taken together, these analyses provide a more comprehensive understanding of the reliability and limitations of the evaluated models.

The error analysis revealed other important insights into the limitations of LLM-based information extraction from clinical text. The predominance of context-related errors underscores the critical role of deep textual comprehension in accurate extraction. This suggests that performance is influenced not only by algorithmic improvements, but also on the linguistic quality and structural clarity of clinical documentation, highlighting the potential benefit of promoting clearer and more standardized practices in electronic health records. Furthermore, the presence of contradictory information as a source of misclassification likely reflects a limitation of our study design, since clinical notes from different encounters were concatenated into a single text for analysis, which may have introduced temporal inconsistencies or conflicting statements within the same input.

### Limitations

This study has some limitations. First, the reference benchmark was created by human reviewers without inter-rater agreement testing. However, the annotated variables corresponded to simple and objective clinical attributes (e.g., smoking status, fever, myocardial infarction), which involve limited interpretative subjectivity. Therefore, the absence of concordance metrics is unlikely to have introduced significant inconsistency in the gold standard. Nevertheless, future studies would benefit from including multiple annotators and reporting agreement statistics, especially for more complex or context-dependent clinical variables. Second, the evaluation focused on cardiology and infectiology outpatient notes, which may limit generalizability to other specialties. Third, more recent high-parameter models (e.g., GPT-5, Claude Opus, Gemini) were not assessed and may demonstrate superior performance. Fourth, the use of quantized model versions to enhance computational efficiency may have introduced performance degradation, particularly in tasks requiring fine-grained semantic interpretation. Some clinical variables, such as “tumors” or “skin lesions”, were intentionally defined in broad terms to reflect real-world documentation practices, in which benign and malignant neoplasms are often not distinguished. While this approach increases sensitivity, it introduces semantic heterogeneity and limits direct clinical applicability. Future research may explore automated subclassification using clinical taxonomies such as SNOMED-CT (Systematized Nomenclature of Medicine - Clinical Terms) or ICD-10 (10th revision of the International Classification of Diseases) or models specialized in semantic disambiguation. Additionally, we did not include comparisons with classical machine learning approaches (e.g., CRF, Support Vector Machine - SVM, Random Forest with feature engineering) or traditional NLP pipelines. While we focused on evaluating different LLMs under a standardized prompting framework, such comparisons could provide important context to better quantify the real incremental gains of LLM-based methods. Future studies should address this gap by systematically comparing LLMs with classical NLP techniques in Portuguese-language clinical corpora. Fifth, regional spelling variation in Brazilian Portuguese was not assessed. Lastly, despite being partially supported by multilingual models, Portuguese remains a performance bottleneck in most LLMs predominantly trained on English corpora.

### Clinical and operational implications

Overall, our findings support the use of LLMs for structured data extraction in Portuguese-language clinical narratives, demonstrating that prompt-tuned models can achieve clinically acceptable performance. Applying the best-performing models to the full dataset has the potential to automate patient triage for research, identify condition prevalence, and build clinical cohorts based on structured inclusion criteria. These capabilities can substantially reduce the manual workload associated with chart review, especially in under-resourced healthcare settings. However, the human effort required to validate LLM outputs remains a key scalability barrier. This effort typically occurs within an iterative “human-in-the-loop” framework, which is crucial for prompt refinement and benchmark correction, especially in specialized domains such as medicine. Although this process demands an initial investment of expert time and resources, the subsequent gains in automation and large-scale data processing capacity may justify this upfront effort. A Quality Assurance (QA) process was conducted by researchers and operational hospital staff to assure security and privacy. Future research should explore strategies to optimize this feedback cycle, such as employing active learning techniques to prioritize uncertain or high-impact cases for human review, thereby minimizing annotation burden.

A potential limitation is the use of the same annotated dataset for both prompt construction and model evaluation, which may raise concerns about potential bias and reduced generalizability. However, the prompts were not tailored to specific examples from the dataset, but rather designed to capture general linguistic structures and broad clinical reasoning patterns. The same standardized prompts were applied consistently to all models, without individual adjustments. Nevertheless, future studies should employ independent datasets for prompt development and evaluation to fully eliminate any residual risk of information leakage and to strengthen external validity.

## Conclusion

This study demonstrates that prompt-tuned and quantized LLMs can effectively extract structured data from unstructured Portuguese clinical notes, offering a viable approach for resource-constrained healthcare systems. Despite infrastructural and linguistic challenges, these models achieved reliable performance across many variables, supporting their potential as cost-efficient tools for structured data extraction in low-resource settings. Future research should expand investigations to other specialties, evaluate newer high-parameter models, validate performance across healthcare institutions, and further assess the trade-offs introduced by quantization. To address linguistic heterogeneity and regionalisms characteristic of Brazilian Portuguese medical documentation, future efforts should consider the development of more diverse corpora, incorporating clinical notes from multiple institutions and geographic regions. Fine-tuning LLMs on such datasets could significantly enhance model robustness and generalization. These findings support the use of LLMs for scalable clinical data extraction in non-English and low-resource healthcare systems. Although this study focused on pre-trained LLMs with prompt engineering and quantization, promising future directions include the application of Parameter-Efficient Fine-Tuning (PEFT) techniques. Methods such as Low-Rank Adaptation (LoRA) or Quantized LoRA (QLoRA) could be employed to adapt models to the specific linguistic subdomain of Portuguese clinical notes, potentially improving performance on complex or infrequent variables without incurring the full computational overhead of fine-tuning large-scale models.

## Data Availability

The research data are available upon request to the corresponding author.
