# Supplementary material for: Benchmarking large language models for clinical data extraction from Portuguese medical notes in a university hospital
Source: Cad Saude Publica. 2026 Jul 31;42:e00145025. doi: 10.1590/0102-311XEN145025 (PMC13431991; doi:10.1590/0102-311XEN145025)
Supplement: Supplementary Material [file 1678-4464-csp-42-EN145025-s.pdf]

SUPPLEMENTARY MATERIAL

Prompt Engineering and Model Parameters

To ensure consistent and directly comparable extraction of clinical variables, each model received the same structured prompt template. First, a concise instruction directed the model to fill a predefined JSON schema; this schema was embedded immediately thereafter. Next, the raw clinical note was provided, followed by a bullet-list of exemplary synonyms and real-world examples for each target variable. Placing the JSON at the end of the instruction block guaranteed uniform field order and formatting across responses. Generation settings remained identical for all models—greedy decoding (do_sample=False), up to 256 new tokens. No top-k or nucleus sampling was employed.

All models were utilized in their off-the-shelf form with no additional training or fine-tuning performed. Quantization was applied during loading through the BitsAndBytes library (version 0.45.5), solely to reduce memory footprint during inference. Specifically:

- **Mixtral-8×7B & DeepSeek-V3** were quantized to 4-bit using NF4 with double quantization.
- **Qwen-32B** was quantized to 8-bit integer.
- **LLaMA 8B** remained in full 16-bit precision (float16).

No quantization-aware training or calibration was carried out; the approach relied entirely on structuring the extraction pipeline. Clinical notes were first normalized (lowercased, accent-stripped) and then split into overlapping context windows to respect each model's input token limit. These preprocessing steps ensured stable performance without modifying the pretrained weights.

Supplementary Tables

Table S1: Key-terms for cardiology variables.

Variables	Key-terms in Portuguese
Tabagism	**Absence (0):** nega tabagismo; não fuma; nega tbg.<br>**Presence (1):** fumante; tabagista; tabagismo; tbg; ex tabagista; ex tabagismo; ex fumante; passado de tabagismo.
Hypertension	**Absence (0):** pa adequada; pressão arterial adequada; nega has; nega hipertensão.<br>**Presence (1):** has; hipertensão arterial; hipertensão arterial sistêmica; hipertenso; hipertensa; pressão arterial elevada; pa elevada; hipertensão.
Diabetes	**Absence (0):** nega diabetes; nega dm; nega diabetes mellitus; nega diabete melito; nega dm2.<br>**Presence (1):** dm; dm2; dm 2; dm tipo 2; dm tipo ii; diabetes mellitus; diabetes melito; diabetes; diabético; diabética; pré-diabetes; intolerância à glicose; disglicemia; insulina; metformina; glicemia de jejum alterada; glifage.
Dyslipidemia	**Absence (0):** nega dislipidemia; nega hipercolesterolemia; nega hipertrigliceridemia; perfil lipídico normal.<br>**Presence (1):** dislipidêmico; dislipidêmica; dislipidemia; dlp; hipertrigliceridemia; hipercolesterolemia;

	hiperlipidemia; uso de estatina; rosuvastatina; sinvastatina; atorvastatina; ezetimibe; ciprofibrato.
Family history	**Absence (0):** nega dm na família; nega avc na família; nega has na família; nega dlp na família; nega história familiar de dm; nega história familiar de has; nega história familiar de dlp; nega história familiar de avc; nega antecedente familiar de dm; nega antecedente familiar de has; nega antecedente familiar de avc; nega antecedente familiar de dlp.<br>**Presence (1):** mãe hipertensa; mãe diabética; mãe dislipidêmica; mãe avc; pai hipertenso; pai diabético; pai dislipidêmico; pai avc; história familiar de hipertensão; história familiar de has; história familiar de diabetes; historia familiar de dm; história familiar de dislipidemia; história familiar de dlp; antecedente familiar de hipertensão; antecedente familiar de has; antecedente familiar de diabetes; antecedente familiar de dm; antecedente familiar de dislipidemia; antecedente familiar de dlp; história familiar de avc; antecedente familiar de avc.
Family history of CAD	**Absence (0):** nega história familiar de dac; nega história familiar de iam; nega história familiar de infarto; nega antecedente familiar de dac; nega antecedente familiar de iam; nega antecedente familiar de infarto; nega dac na família; nega iam na família<br>**Presence (1):** mãe com dac; pai com dac; mãe teve iam; pai teve iam.
Angina	**Absence (0):** assintomático; nega sintomas cardiovasculares; sem queixas; sem queixas cv; nega angina; nega dor torácica; nega dor precordial; nega precordialgia; dor torácica não anginosa; dor torácica não

	cardíaca; dor torácica musculoesquelética.<br>**Presence (1):** angina; dor precordial; precordialgia; dor torácica; angina estável; angina instável; dor no peito.
Myocardial infarction	**Absence (0):** nega infarto; nega iam; nega dac.<br>**Presence (1):** refere infarto prévio; relata infarto prévio; iam; infarto; iamcsst; iamssst; scacsst; scassst.
Obesity	**Absence (0):** -<br>**Presence (1):** obeso; obesa; obesidade.
Sedentarism	**Absence (0):** -<br>**Presence (1):** sedentário; sedentária; sedentarismo; não realiza atividades físicas.
Stroke	**Absence (0):** nega avc.<br>**Presence (1):** refere avc prévio; relata avc prévio; avc; avci; avch; ave.
Aspirin use	**Absence (0):** -<br>**Presence (1):** aspirina; aas; somalgin cárdio.
Atrial fibrillation	**Absence (0):** nega fibrilação atrial; nega fa; ritmo sinusal<br>**Presence (1):** fibrilação atrial; fa.
Heart failure	**Absence (0):** nega insuficiência cardíaca; nega ic; nega icc.<br>**Presence (1):** insuficiência cardíaca; ic; icc; icfer; icfep; icfei; icfem; icfelr.

Table S2: Key-terms for infectiology variables.

Variables	Key-terms in Portuguese
Fever	**Absence (0):** afebril; apirético; nega febre; nega quadro febril.<br><br>**Presence (1):** refere febre; relata febre; febre; febril; apresenta febre; temperatura elevada
Weight loss	**Absence (0):** nega perda pondera; nega perda de peso.<br><br>**Presence (1):** refere perda ponderal; relata perda ponderal; refere perda de peso; relata perda de peso; emagrecida; emagrecido; síndrome consumptiva.
Cough	**Absence (0):** nega tosse; ausência de sintomas respiratórios; sem sintomas respiratórios.<br><br>**Presence (1):** refere tosse; relata tosse; síndrome gripal; queixa de tosse.
Diarrhea	**Absence (0):** nega diarreia; ritmo intestinal normal; ritmo intestinal habitual; dejeções sem alterações; nega alteração do ritmo intestinal; dejeções preservadas.<br><br>**Presence (1):** refere diarreia; relata diarreia; dejeções pastosas; dejeções líquidas; fezes amolecidas; fezes diarreicas.
Jaundice	**Absence (0):** anictérico; anictérica; sem icterícia.<br><br>**Presence (1):** ictérico; ictérica; icterícia.
Pain	**Absence (0):** nega dor; nega quadros álgicos; sem dor; sem queixas álgicas; sem quadros álgicos.<br><br>**Presence (1):** refere dor; relata dor; mantém dor; mantendo dor; evoluiu com dor; apresenta dor;

	apresenta quadro álgico; apresenta queixas álgicas; dor crônica.
Seizures	**Absence (0):** nega convulsão; nega crise epiléptica; sem crise epiléptica.<br>**Presence (1):** histórico de convulsão; histórico de crise convulsiva; histórico de crise epiléptica; apresentou convulsão; apresentou crise convulsiva; apresentou crise epiléptica; refere convulsão; relata convulsão; epilepsia.
Skin spots	**Absence (0):** nega alterações cutâneas; nega alterações na pele; sem lesões de pele; pele sem alteração; pele íntegra.<br>**Presence (1):** manchas hipocrômicas; manchas hipercrômicas; manchas acrômicas; manchas na pele; lesões na pele; lesões descamativas; lesões bolhosas; ferida; furúnculo; verruga; máculas; pápulas; manchas; escaras; lesões vesiculares; vesículas; lesões pruriginosas; placa eritematosa; melasma; furunculose; úlcera; crostas; lesão vegetante; ferimento; rash cutâneo; exantema.
Tumors	**Absence (0):** nega câncer; sem neoplasias; afastado câncer; afastados neoplasias.<br>**Presence (1):** carcinoma; adenoma; adenocarcinoma; neoplasia; mioma; sarcoma; câncer; linfoma.
Dyspnea	**Absence (0):** nega falta de ar; nega dispneia; sem dispneia; sem falta de ar.<br>**Presence (1):** dispneico; dispneica; dispneia; falta de ar.
Hyposmia/anosmia	**Absence (0):** nega alterações de olfato; nega alterações de paladar; nega anosmia; nega ageusia; nega hiposmia.

	**Presence (1):** anosmia; hiposmia; ageusia; perda de paladar; perda de olfato; disgeusia.
Illicit drug use	**Absence (0):** nega uso de drogas ilícitas; nega uso de substâncias; nega droga ilícita; não utiliza drogas ilícitas; nega uso de outras drogas.<br>**Presence (1):** uso de drogas ilícitas; uso de drogas; usuária de drogas; usuário de drogas; relata consumo de drogas; relata consumo de substâncias; uso de substâncias.
Diabetes	**Absence (0):** nega diabetes; nega dm; nega diabetes mellitus; nega diabete melito; nega dm2.<br>**Presence (1):** dm; dm2; dm 2; dm tipo 2; dm tipo ii; diabetes mellitus; diabetes melito; diabetes; diabético; diabética; pré-diabetes; intolerância à glicose; disglicemia; insulina; metformina; glicemia de jejum alterada; glifage.
Hypertension	**Absence (0):** pa adequada; pressão arterial adequada; nega has; nega hipertensão.<br>**Presence (1):** has; hipertensão arterial; hipertensão arterial sistêmica; hipertenso; hipertensa; pressão arterial elevada; pa elevada; hipertensão.

Table S3: F1-score for the cardiology dataset

Variable	GPT-4o mini	DeepSeek-V3	Mixtral-8x7B	LLaMA 8B	Qwen-32B	Mean
Tabagism	0.57	0.62	0.51	**0.84**	*0.71*	0.65
Hypertension	**0.95**	0.90	0.89	0.90	*0.94*	0.92
Diabetes	*0.91*	0.74	0.90	0.88	**0.94**	0.87
Dyslipidemia	0.86	0.85	0.84	*0.89*	**0.91**	0.87
Family history	**0.89**	0.48	0.62	0.41	*0.83*	0.65
Family history of CAD	*0.75*	0.51	0.44	0.34	**0.82**	0.57
Angina	0.85	0.72	**0.86**	0.74	**0.86**	0.80
Myocardial infarction	*0.73*	0.63	0.53	0.42	**0.81**	0.62
Obesity	0.64	0.73	**0.84**	0.64	*0.83*	0.74
Sedentarism	0.57	*0.74*	0.61	0.71	**0.89**	0.70
Stroke	**0.91**	0.67	0.67	0.43	*0.83*	0.70
Aspirin use	**0.93**	0.72	0.32	0.62	*0.90*	0.70
Atrial fibrillation	0.58	**0.75**	0.71	0.43	*0.73*	0.64
Heart failure	0.54	*0.78*	**0.85**	0.68	0.70	0.71

Mean (SD)	*0.76 (0.15)*	0.70 (0.11)	0.69 (0.18)	0.64 (0.19)	**0.83 (0.08)**

CAD: coronary artery disease; SD: standard deviation. The model with the best performance is highlighted in **bold**, and the second in *italics*.

Table S4: Recall (sensitivity) for the cardiology dataset

Variable	GPT-4o mini	DeepSeek-V3	Mixtral-8x7B	LLaMA 8B	Qwen-32B	Mean
Tabagism	0.41	0.50	0.34	**0.91**	*0.56*	0.54
Hypertension	0.99	0.98	0.99	**1.00**	**1.00**	0.99
Diabetes	**0.97**	0.96	0.87	0.96	0.96	0.95
Dyslipidemia	0.94	0.95	0.87	**1.00**	*0.97*	0.95
Family history	**1.00**	0.97	0.97	**1.00**	**1.00**	0.99
Family history of CAD	0.91	0.91	0.38	**1.00**	*0.95*	0.83
Angina	0.93	0.62	0.91	*0.95*	**0.96**	0.87
Myocardial infarction	**0.96**	0.84	0.52	0.92	0.92	0.83
Obesity	**1.00**	0.96	0.96	0.96	**1.00**	0.98
Sedentarism	**1.00**	0.97	**1.00**	**1.00**	**1.00**	0.99
Stroke	*0.91*	0.76	0.52	**0.95**	0.81	0.79
Aspirin use	0.98	0.93	0.20	0.95	**1.00**	0.81
Atrial fibrillation	**0.95**	*0.91*	0.71	0.86	*0.91*	0.87
Heart failure	*0.98*	0.78	0.95	0.95	**1.00**	0.93

Mean (SD)	0.92 (0.15)	0.86 (0.14)	0.73 (0.27)	**0.96 (0.04)**	*0.93 (0.12)*

CAD: coronary artery disease; SD: standard deviation. The model with the best performance is highlighted in **bold**, and the second in *italics*.

Table S5: F1-score for the infectiology dataset

Variable	GPT-4o mini	DeepSeek-V3	Mixtral-8x7B	LLaMA 8B	Qwen-32B	Mean
Fever	**0.90**	0.22	0.73	0.74	*0.86*	0.69
Weight loss	*0.84*	0.61	0.73	0.62	**0.90**	0.74
Cough	**0.92**	0.76	0.88	0.88	*0.89*	0.87
Diarrhea	**0.90**	0.79	0.72	0.76	**0.90**	0.82
Jaundice	*0.89*	0.74	0.74	0.74	**0.96**	0.81
Pain	*0.91*	0.80	0.85	0.80	**0.93**	0.86
Seizures	0.91	0.78	*0.92*	0.82	**1.00**	0.89
Skin spots	*0.78*	*0.78*	0.77	0.76	**0.82**	0.78
Tumors	*0.72*	0.48	0.65	**0.73**	0.70	0.66
Dyspnea	*0.96*	0.83	0.94	0.80	**0.98**	0.90
Anosmia or ageusia	*0.90*	0.70	0.87	0.83	**0.91**	0.84
Illicit drug use	*0.51*	*0.51*	0.45	0.35	**0.72**	0.51
Diabetes	0.65	0.54	**0.84**	*0.71*	0.69	0.69
Hypertension	0.52	**0.68**	0.57	0.57	*0.64*	0.59

Mean (SD)	*0.81 (0.14)*	0.66 (0.17)	0.76 (0.13)	0.72 (0.13)	**0.85 (0.11)**

SD: standard deviation. The model with the best performance is highlighted in **bold**, and the second in *italics*.

Table S6: Recall (sensitivity) for the infectiology dataset

Variable	GPT-4o mini	DeepSeek-V3	Mixtral-8x7B	LLaMA 8B	Qwen-32B	Mean
Fever	*0.89*	0.13	0.60	**0.92**	0.83	0.67
Weight loss	*0.98*	0.86	0.91	**1.00**	*0.98*	0.95
Cough	*0.90*	0.65	0.83	**0.91**	0.86	0.83
Diarrhea	**1.00**	0.70	0.95	*0.97*	0.89	0.90
Jaundice	0.95	0.62	0.95	**1.00**	**1.00**	0.91
Pain	0.94	0.91	0.83	**1.00**	*0.97*	0.93
Seizures	0.95	0.90	0.90	**1.00**	**1.00**	0.95
Skin spots	**0.83**	0.70	0.67	**0.83**	0.81	0.77
Tumors	**0.92**	0.48	0.56	0.80	*0.84*	0.72
Dyspnea	**1.00**	0.77	**1.00**	**1.00**	**1.00**	0.96
Anosmia or ageusia	**1.00**	0.82	0.94	**1.00**	0.94	0.94
Illicit drug use	**1.00**	0.86	0.95	**1.00**	0.91	0.94
Diabetes	**1.00**	0.90	0.76	*0.93*	*0.93*	0.90
Hypertension	**1.00**	0.75	0.94	0.96	*0.98*	0.93

Mean (SD)	**0.95 (0.05)**	0.72 (0.20)	0.84 (0.14)	**0.95 (0.07)**	0.92 (0.07)

SD: standard deviation. The model with the best performance is highlighted in **bold**, and the second in *italics*.

Table S7: Paired Performance Comparison Between Language Models (McNemar Test) for the Cardiology Dataset

Variable	GPT-4o mini VS DeepSeek-V3	GPT-4o mini VS LLaMA 8B	GPT-4o mini VS Mixtral-8x7B	GPT-4o mini VS Qwen-32B	DeepSeek-V3 VS LLaMA 8B	DeepSeek-V3 VS Mixtral-8x7B	DeepSeek-V3 VS Qwen-32B	LLaMA 8B VS Mixtral-8x7B	LLaMA 8B VS Qwen-32B	Mixtral-8x7B VS Qwen-32B
**Tabagism**	1.000	0.108	1.000	0.063	0.108	1.000	0.267	0.076	0.523	**<0.001**
**Obesity**	**0.041**	1.000	**<0.001**	**<0.001**	0.078	**0.039**	0.118	**<0.001**	**0.002**	1.000
**Aspirin use**	**<0.001**	**<0.001**	**<0.001**	0.388	**<0.001**	0.567	**<0.001**	0.089	**<0.001**	**<0.001**
**Angina**	0.108	**0.001**	1.000	1.000	0.184	0.064	0.099	**<0.001**	**<0.001**	1.000
**Myocardial infarction**	0.210	**<0.001**	0.458	0.167	**<0.001**	0.851	**0.003**	**<0.001**	**<0.001**	**0.036**
**Family history**	**<0.001**	**<0.001**	**<0.001**	**0.031**	**<0.001**	**<0.001**	**<0.001**	**<0.001**	**<0.001**	**0.000**
**Dyslipidemia**	0.503	0.070	0.481	**0.004**	**0.021**	1.000	**0.002**	0.052	0.180	**0.001**
**Stroke**	**0.002**	**<0.001**	0.065	0.375	**<0.001**	0.332	**0.035**	**<0.001**	**<0.001**	0.344
**Heart failure**	**<0.001**	**<0.001**	**<0.001**	**<0.001**	**0.002**	0.503	**0.005**	**<0.001**	1.000	**<0.001**
**Sedentarism**	**<0.001**	**0.001**	0.392	**<0.001**	0.585	**0.002**	**0.007**	**0.004**	**<0.001**	**<0.001**
**Diabetes**	**<0.001**	0.180	1.000	0.227	**<0.001**	**<0.001**	**<0.001**	0.286	**0.019**	0.332
**Atrial fibrillation**	**0.002**	**0.002**	**0.002**	**0.004**	**<0.001**	1.000	1.000	**<0.001**	**<0.001**	0.774
**Family**	**<0.001**	**<0.001**	0.210	0.388	**<0.001**	**0.020**	**<0.001**	**<0.001**	**<0.001**	**0.035**

**history of CAD**										
**Hypertension**	**0.001**	**0.001**	**<0.001**	0.549	1.000	1.000	**0.002**	0.500	**0.003**	**<0.001**

Bold values indicate statistically significant differences ($p < 0.05$).

Table S8: Paired Performance Comparison Between Language Models (McNemar Test) for the Infectology Dataset

Variable	GPT-4o mini VS DeepSeek-V3	GPT-4o mini VS LLaMA 8B	GPT-4o mini VS Mixtral-8x7B	GPT-4o mini VS Qwen-32B	DeepSeek-V3 VS LLaMA 8B	DeepSeek-V3 VS Mixtral-8x7B	DeepSeek-V3 VS Qwen-32B	LLaMA 8B VS Mixtral-8x7B	LLaMA 8B VS Qwen-32B	Mixtral-8x7B VS Qwen-32B
Fever	**<0.001**	**<0.001**	**0.008**	0.344	0.130	**<0.001**	**<0.001**	0.200	**0.003**	0.115
Weight loss	**<0.001**	**<0.001**	**0.009**	0.180	0.451	**0.010**	**<0.001**	**<0.001**	**<0.001**	**<0.001**
Cough	**0.001**	0.180	0,219	0.375	0.108	**0.031**	**0.019**	1.000	0.774	1.000
Diarrhea	0.210	**0.001**	**<0.001**	1.000	0.163	**0.035**	0.092	0.557	**0.001**	**<0.001**
Jaundice	0.344	**0.013**	**0.022**	0.250	0.286	0.359	0.065	1.000	**<0.001**	**<0.001**
Pain	**<0.001**	**<0.001**	**0.015**	0.424	0.327	**0.027**	**<0.001**	**0.004**	**<0.001**	**0.005**
Seizures	0.146	0.125	1.000	0.125	1.000	0.065	**0.002**	0.109	**0.004**	0.250
Skin spots	0.572	0.557	0.572	0.167	0.229	1.000	0.678	0.215	0.061	0.664
Tumors	0.185	0.607	0.664	1.000	**0.035**	**0.027**	0.169	1.000	0.607	0.648
Dyspnea	0.180	**0.004**	1.000	1.000	0.481	0.289	0.070	**0.021**	**0.002**	0.500
Anosmia or ageusia	1.000	1.000	1.000	1.000	1.000	1.000	1.000	1.000	1.000	1.000
Illicit drug use	1.000	1.000	1.000	1.000	1.000	1.000	1.000	1.000	1.000	1.000
Diabetes	**0.005**	**0.029**	**<0.001**	0.110	**<0.001**	**<0.001**	**<0.001**	**0.001**	0.728	**<0.001**
Hypertension	**<0.001**	**0.006**	**0.005**	**<0.001**	**<0.001**	**<0.001**	**0.010**	1.000	**0.006**	**0.014**

Bold values indicate statistically significant differences ($p < 0.05$).

Table S9: Bootstrap Estimates of Model Performance for the Cardiology Dataset

Variable	GPT-4o mini	DeepSeek-V3	Mixtral-8x7B	LLaMA 8B	Qwen-32B
Tabagism	0.70 (0.62 – 0.78)	0.73 (0.64 – 0.82)	0.67 (0.59 – 0.75)	**0.92 (0.86 – 0.97)**	*0.78 (0.70 – 0.87)*
Hypertension	**0.81 (0.72 – 0.89)**	0.59 (0.52 – 0.66)	0.56 (0.51 – 0.62)	0.58 (0.52 – 0.64)	*0.75 (0.67 – 0.84)*
Diabetes	*0.90 (0.86 – 0.95)*	0.65 (0.59 – 0.71)	0.90 (0.85 – 0.95)	0.86 (0.81 – 0.92)	**0.94 (0.90 – 0.97)**
Dyslipidemia	0.70 (0.62 – 0.77)	0.65 (0.57 – 0.71)	0.70 (0.63 – 0.78)	*0.72 (0.65 – 0.80)*	**0.81 (0.73 – 0.87)**
Family history	**0.96 (0.94 – 0.98)**	0.66 (0.61 – 0.72)	0.80 (0.75 – 0.85)	0.56 (0.53 – 0.58)	*0.94 (0.91 – 0.96)*
Family history of CAD	*0.91 (0.83 – 0.97)*	0.83 (0.75 – 0.89)	0.66 (0.57 – 0.77)	0.69 (0.65 – 0.73)	**0.95 (0.89 – 0.99)**
Angina	*0.89 (0.84 – 0.94)*	0.78 (0.71 – 0.84)	*0.89 (0.84 – 0.94)*	0.80 (0.74 – 0.86)	**0.91 (0.86 – 0.95)**

Myocardial infarction	*0.91 (0.86 – 0.96)*	0.84 (0.75 – 0.91)	0.72 (0.62 – 0.81)	0.72 (0.65 – 0.79)	**0.93 (0.86 – 0.98)**
Obesity	0.90 (0.86 – 0.93)	0.92 (0.87 – 0.96)	*0.95 (0.90 – 0.98)*	0.88 (0.82 – 0.93)	**0.96 (0.94 – 0.98)**
Sedentarism	0.81 (0.77 – 0.85)	*0.90 (0.85 – 0.95)*	0.84 (0.79 – 0.88)	*0.90 (0.86 – 0.94)*	**0.97 (0.94 – 0.99)**
Stroke	**0.94 (0.87 – 1.00)**	0.84 (0.74 – 0.93)	0.76 (0.64 – 0.88)	0.78 (0.71 – 0.83)	*0.90 (0.80 – 0.97)*
Aspirin use	**0.96 (0.92 – 0.99)**	0.78 (0.73 – 0.84)	0.59 (0.54 – 0.65)	0.67 (0.61 – 0.72)	*0.94 (0.90 – 0.97)*
Atrial fibrillation	0.87 (0.81 – 0.92)	**0.91 (0.84 – 0.97)**	0.83 (0.73 – 0.93)	0.76 (0.67 – 0.84)	**0.91 (0.83 – 0.97)**
Heart failure	0.70 (0.64 – 0.75)	*0.85 (0.79 – 0.92)*	**0.92 (0.88 – 0.96)**	0.82 (0.77 – 0.87)	0.84 (0.80 – 0.88)

CAD: coronary artery disease. The results are expressed in mean (percentile 2.5 – percentile 97.5). The model with the best performance is highlighted in **bold**, and the second in *italics*.

Table S10: Bootstrap Estimates of Model Performance for the Cardiology Dataset

Variable	GPT-4o mini	DeepSeek-V3	Mixtral-8x7B	LLaMA 8B	Qwen-32B
Fever	**0.93 (0.88 – 0.97)**	0.56 (0.51 – 0.61)	0.79 (0.71 – 0.86)	0.85 (0.80 – 0.90)	*0.89 (0.84 – 0.94)*
Weight loss	*0.93 (0.89 – 0.96)*	0.77 (0.70 – 0.84)	0.85 (0.80 – 0.90)	0.79 (0.75 – 0.83)	**0.95 (0.92 – 0.98)**
Cough	**0.93 (0.89 – 0.97)**	0.81 (0.75 – 0.87)	0.90 (0.85 – 0.95)	***0.92 (0.87 – 0.96)***	0.89 (0.84 – 0.95)
Diarrhea	**0.97 (0.95 – 0.99)**	0.84 (0.77 – 0.92)	0.88 (0.83 – 0.92)	0.91 (0.87 – 0.95)	*0.93 (0.87 – 0.98)*
Jaundice	*0.96 (0.91 – 0.99)*	0.81 (0.69 – 0.90)	0.93 (0.87 – 0.97)	0.95 (0.92 – 0.97)	**0.99 (0.98 – 1.00)**
Pain	*0.85 (0.80 – 0.91)*	0.62 (0.56 – 0.69)	0.79 (0.73 – 0.86)	0.53 (0.51 – 0.57)	**0.87 (0.81 – 0.92)**
Seizures	0.97 (0.91 – 1.00)	0.92 (0.85 – 0.98)	0.95 (0.87 – 1.00)	*0.97 (0.95 – 0.99)*	**1.00 (1.00 – 1.00)**

Skin spots	*0.82 (0.76 – 0.87)*	*0.82 (0.75 – 0.88)*	0.81 (0.75 – 0.87)	0.80 (0.74 – 0.86)	**0.86 (0.80 – 0.91)**
Tumors	**0.91 (0.84 – 0.96)**	0.70 (0.60 – 0.80)	0.77 (0.67 – 0.87)	0.86 (0.78 – 0.94)	*0.88 (0.80 – 0.95)*
Dyspnea	*0.99 (0.98 – 1.00)*	0.88 (0.79 – 0.95)	*0.99 (0.98 – 1.00)*	0.96 (0.94 – 0.98)	**1.00 (0.99 – 1.00)**
Anosmia or ageusia	**0.99 (0.97 – 1.00)**	0.88 (0.79 – 0.97)	0.96 (0.90 – 1.00)	*0.98 (0.96 – 0.99)*	0.97 (0.90 – 1.00)
Illicit drug use	0.40 (0.32 – 0.51)	*0.50 (0.40 – 0.62)*	**0.51 (0.39 – 0.64)**	0.45 (0.32 – 0.57)	0.46 (0.39 – 0.55)
Diabetes	0.83 (0.79 – 0.87)	0.73 (0.67 – 0.78)	**0.87 (0.80 – 0.93)**	*0.86 (0.80 – 0.91)*	0.85 (0.80 – 0.90)
Hypertension	0.62 (0.59 – 0.66)	**0.78 (0.71 – 0.85)**	0.68 (0.63 – 0.73)	0.68 (0.63 – 0.73)	*0.77 (0.71 – 0.82)*

The results are expressed in mean (percentile 2.5 – percentile 97.5). The model with the best performance is highlighted in **bold**, and the second in *italics*.
